# Manipulating magnetoelectric properties by interfacial coupling in La_0.3_Sr_0.7_MnO_3_/Ba_0.7_Sr_0.3_TiO_3_ superlattices

**DOI:** 10.1038/s41598-017-08260-y

**Published:** 2017-08-09

**Authors:** Haizhong Guo, Qingqing Li, Zhengzhong Yang, Kui-juan Jin, Chen Ge, Lin Gu, Xu He, Xiaolong Li, Ruiqiang Zhao, Qian Wan, Jiesu Wang, Meng He, Can Wang, Huibin Lu, Yuping Yang, Guozhen Yang

**Affiliations:** 1 Institute of Physics, Chinese Academy of Sciences, Beijing, 100190 China; 2 School of Science, Minzu University of China, Beijing, 100081 China; 3 Collaborative Innovation Center of Quantum Matter, Beijing, China; 4 University of Chinese Academy of Sciences, Beijing, 100049 China; 5 Shanghai Synchrotron Radiation Facility (SSRF), Shanghai Institute of Applied Physics, Chinese Academy of Sciences, Shanghai, 201204 China; 6 School of Physical Engineering, Zhengzhou University, Zhengzhou, Henan 450001 China

## Abstract

Artificial superlattices constructed with ferromagnetic La_0.7_Sr_0.3_MnO_3_ layer and ferroelectric Ba_0.7_Sr_0.3_TiO_3_ layer were designed and fabricated on SrTiO_3_ substrates. An epitaxial growth with sharp interfaces between La_0.7_Sr_0.3_MnO_3_ and Ba_0.7_Sr_0.3_TiO_3_ layers was confirmed by scanning transmission electron microscopy and x-ray diffraction. An unambiguous charge transfer involving an electron transferring from the La_0.7_Sr_0.3_MnO_3_ layers to Ba_0.7_Sr_0.3_TiO_3_ layers (Mn^3+^→Mn^4+^; Ti^4+^→Ti^3+^) across the interface were resolved by electron energy loss spectra analysis. These observations are attributed to the possible modification in the stereochemistry of the Ti and Mn ions in the interfacial region. The out-of-plane lattice parameter, Curie temperature, and magnetoresistance are strongly affected by the thicknesses of the La_0.7_Sr_0.3_MnO_3_ and Ba_0.7_Sr_0.3_TiO_3_ layers. Huge magnetoresistance subsisting to low temperature was also observed in the La_0.7_Sr_0.3_MnO_3_/Ba_0.7_Sr_0.3_TiO_3_ superlattices. All spectral changes identified at a nanometer scale and their potential effect on the degradation of magnetic and transport properties at a macroscopic level. These findings highlight the importance of dependence on sublayer thickness, illustrating the high degree of tenability in these artificially low-dimensional oxide materials.

## Introduction

Controlled interfaces between complex oxide systems with the perovskite oxides, lead to a variety of novel and unexpected electronic and magnetic phenomena and functionalities at the interface that are absent in the respective bulk materials^1^. Representative aspects of recent finding include a two-dimensional electron gas with magnetic and superconducting ground states at strong electric insulating interfaces between SrTiO_3_ and LaAlO_3_^2–5^, emergent ferromagnetism at antiferromagnetic interfaces between LaMnO_3_/SrMnO_3_^6, 7^, interfacial orbital and spin polarization at the interface of a superconductor YBa_2_CuO_7_ and a ferromagnet La_0.7_Ca_0.3_MnO_3_^8, 9^, orbital reconstruction at the interfaces of LaNiO_3_ and LaAlO_3_^10, 11^, and enhanced polarization in BaTiO_3_/SrTiO_3_/CaTiO_3_ superlattice^12, 13^, *etc*. These results also generated an intense debased on the origin of the unexpected properties at the interfaces. However, the competition between multiple order constants induces a great challenge on our ability to predict the resulting novel electronic phases and functionalities and thus to create artificial materials with meet specific application requirements, and these artificial materials in the form of either composites, superlattices, or multilayers. Recently, the superlattices consisting of alternating ferroelectric and ferromagnetic layers yielded unusual electrical and magnetic transport properties that cannot be obtained in either of their constituents, such as Pr_0.85_Ca_0.15_MnO_3_/Ba_0.6_Sr_0.4_TiO_3_ superlattice^14^, La_0.7_Ca_0.3_MnO_3_/BaTiO_3_^15^, La_0.75_Sr_0.25_MnO_3_/Ba_0.7_Sr_0.3_TiO_3_ superlattices^16^, LaMnO_3_/SrTiO_3_^17^, La_0.6_Sr_0.4_MnO_3_/0.7Pb(Mg_1/3_Nb_2/3_)O_3_-0.3(PbTiO_3_) superlattice^18, 19^, etc. However, most of the research was devoted to evaluate the relationship between the whole magnetic and transport properties and individual superlattice layers quality, and it was proposed that these unusual electrical and magnetic transport properties are attributed to the ferroelectric spacer layer and the associated magnetoelectric coupling with the ferromagnetic layer^14–19^. The microscopic structure of the layers and interfaces (e.g. residual strain, strain gradient, atoms arrangement etc.) and electric structures (valence of the ions and charge transfer at the interfaces) should play crucial roles. One the other hand, oxygen vacancies seem to play a key role in these structures, but the puzzle is far from being understood^20^.

Mixed-valence manganite La_0.7_Sr_0.3_MnO_3_ exhibits fantastic rich variety of attractive properties, such as colossal magnetoresistance (MR), a Curie temperature above room temperature (*T*_*C*_ ~ 360 K), half-metallicity, high conductivity, and low coercive field^21–23^. While Ba_0.7_Sr_0.3_TiO_3_ as good a candidate for application in high-density dynamic random access memories and microwave devices is due to their high relative dielectric constant (ε_r_) values and tunable ε_r_ by applying an electric field^24^. In this work, we have grown the artificial superlattices consisting with a ferromagnetic La_0.7_Sr_0.3_MnO_3_ (LSMO) layer and ferroelectric Ba_0.7_Sr_0.3_TiO_3_ (BSTO) layer on (100) SrTiO_3_ (STO) substrates, which are of special relevance in practical dielectric and spintronics devices. The aim in this work is to investigate the crystalline microstructure quality and the electrical structures of the individual layers and interfaces of the LSMO/BSTO superlattices (SLs), and to evaluate their effect on the magnetic and electrical properties and the associated magnetoelectric coupling of the LSMO/BSTO superlattices.

## Results

### Epitaxial nature and crystallinity revealed by SXRD

**Figure 1:**
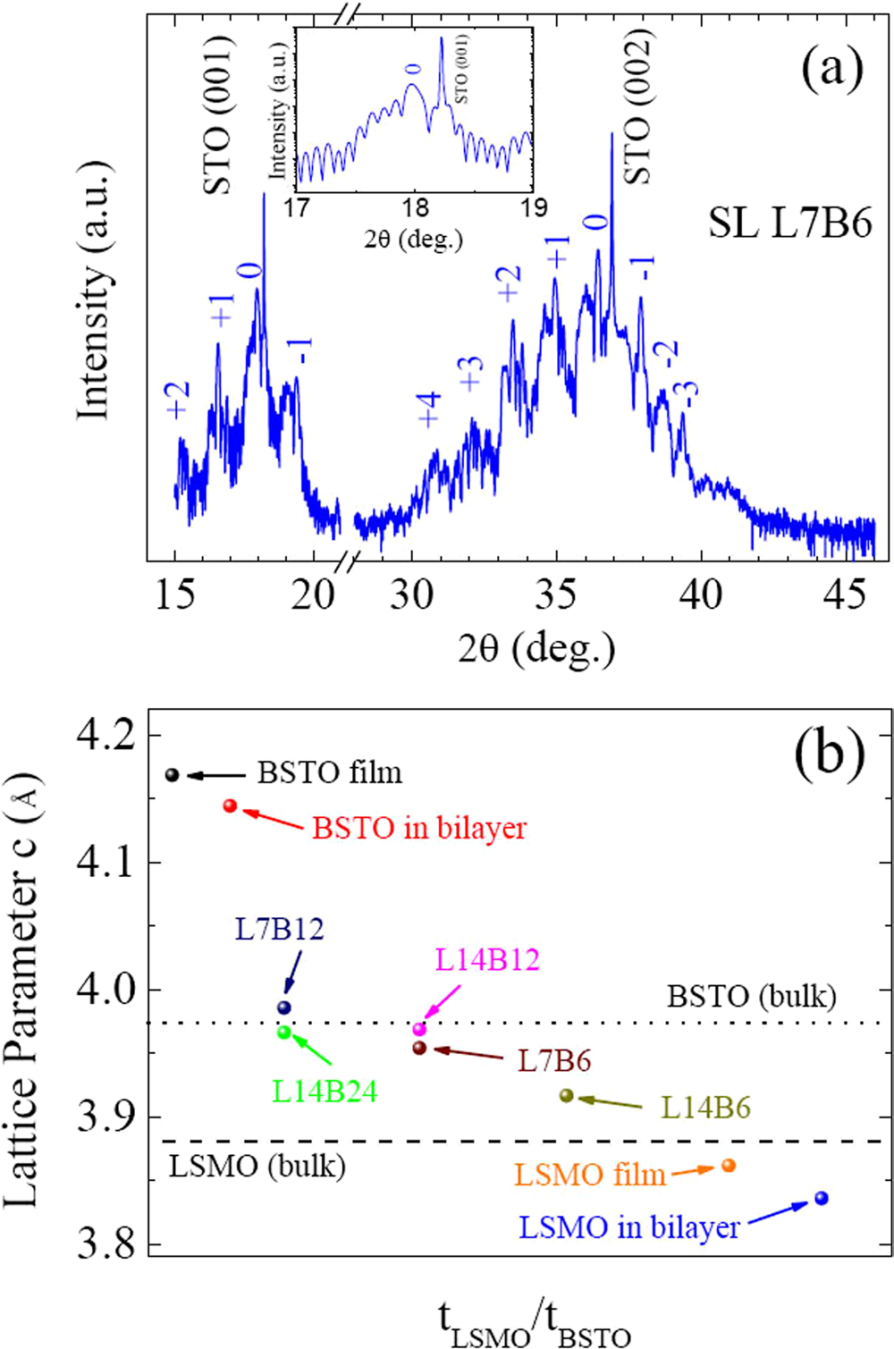
(**a**) SXRD *θ*-2*θ* scan curve of the SL L7B6. The numbers 1, 2, 3,…indicate the order of the satellite peaks. (**b**) The out-of-plane lattice parameter *c* of the superlattices, bilayer, thin films with the thicknesses of the LSMO and BSTO layers. The dished and dotted lines represent the *c*-axis lattice parameter of bulk LSMO and BSTO, respectively. Inset in (**a**) shows Pendellösung fringes around (001) diffraction peak of STO.

**Figure 2:**
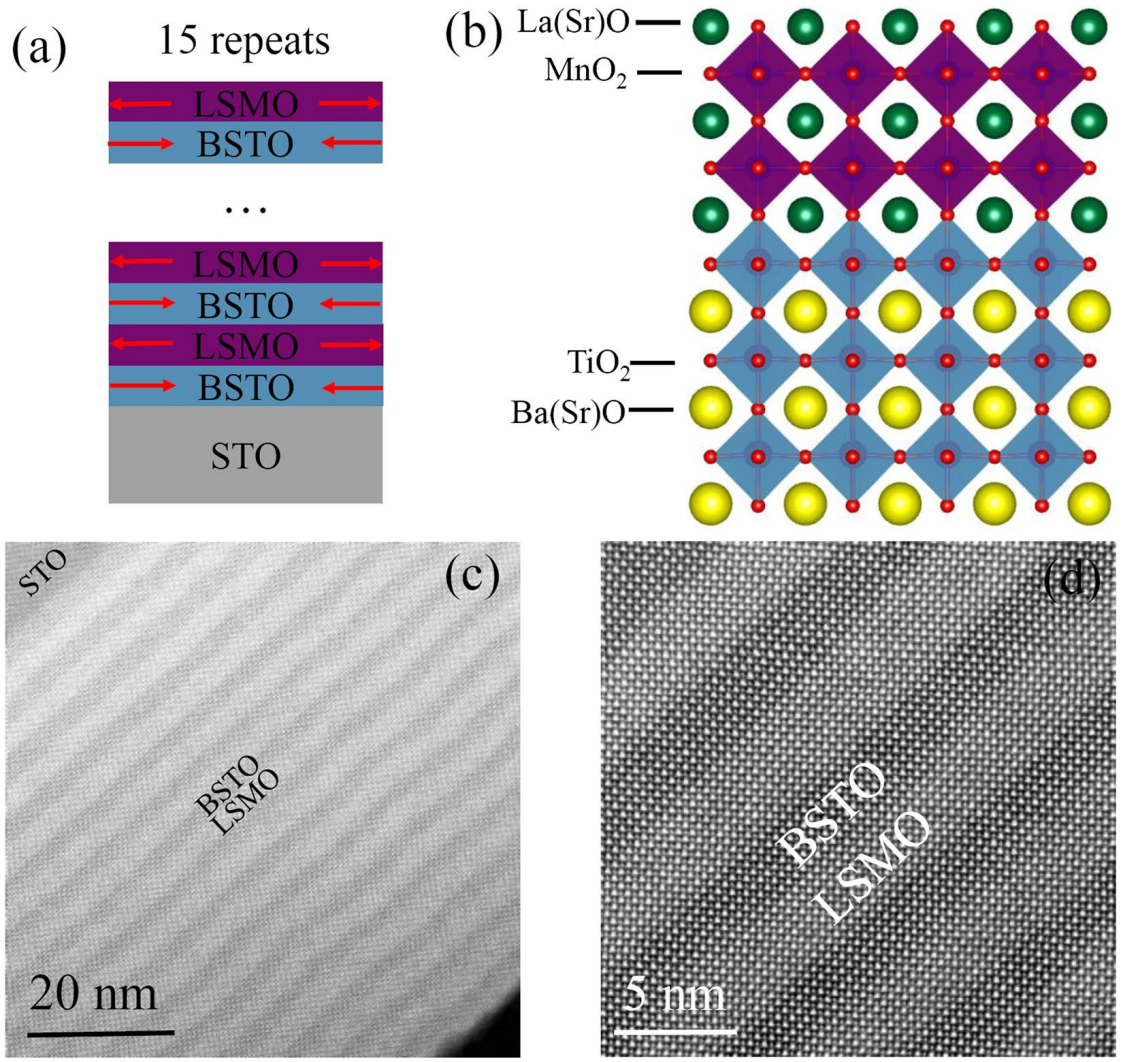
The atomic structure of SL L7B6 grown on STO substrate characterized by STEM. (**a**) The stacking sequence of the LSMO/BSTO SL, showing a single supercell that is repeated the indicated number of times. The red arrows showing the tensile and compress stress along in-plane directions. (**b**) Structure model of LSMO/BSTO SL. (**c**) A low-magnification cross-sectional HAADF-STEM image exhibiting the entire LSMO and BSTO bands and the STO substrate along the [100] axis of STO. (**d**) High-resolution HAADF-STEM image clearly showing the 7-u.c.-thick LSMO band and 6-u.c.-thick BSTO band. Sharp interfaces and lateral coherency also clearly exhibiting. The lighter bands are LSMO, the darker bands BSTO.

The LSMO/BSTO SLs composed of individual LSMO layers with 7 and 14 unit cells (u.c.) and BSTO layer thickness of 6, 12, and 24 u.c. with a total periodicity of 15 were designed and fabricated. The LSMO layers with 7 and 14 u.c. were chosen because the dead layer of LSMO is just below 7 u.c.^25–27^. Figure 1S shows the high-resolution synchrotron x-ray diffractometry (SXRD) results of the various samples, including the LSMO/BSTO SLs, the LSMO and BSTO films, and the LSMO/BSTO/STO bilayers. It can be seen from Fig. 1S that all samples show only the (00 l) Bragg reflections, and no diffraction peaks from secondary phase or randomly oriented grains are observed, indicating that all the films, bilayers, and superlattice were epitaxially grown along the *c*-axis orientation with a good single phase. The SXRD pattern of the SL [LSMO_7_/BSTO_6_]_15_ (L7B6) around (001) and (002) reflections was exhibited in Fig. 1(a). The presence of higher-order satellite peaks (1, 2, 3…) in the LSMO/BSTO SL, which arising from the periodic chemical modulation of multilayer structures, clearly provides evidence for the structural coherency and the good crystalline quality of the LSMO/BSTO SLs. The high quality of the LSMO/BSTO SL can be appreciated from oscillations of integrated x-ray intensity, known as Pendellösung fringes, as shown in the inset of Fig. 1(a). X-ray ϕ scan was carried out to confirm the epitaxial growth of SLs. The (202) ϕ scan result of L14B12 is plotted in Fig. 2S, and four equally spaced peaks separated by 90° were observed, indicating that L14B12 was epitaxially grown on the ST0 substrate. Moreover, the SL peaks are the same with respect to the substrate peaks, suggesting the epitaxial relationships: [001]SL//[001]STO and [100]SL//[100]STO.

**Figure 3:**
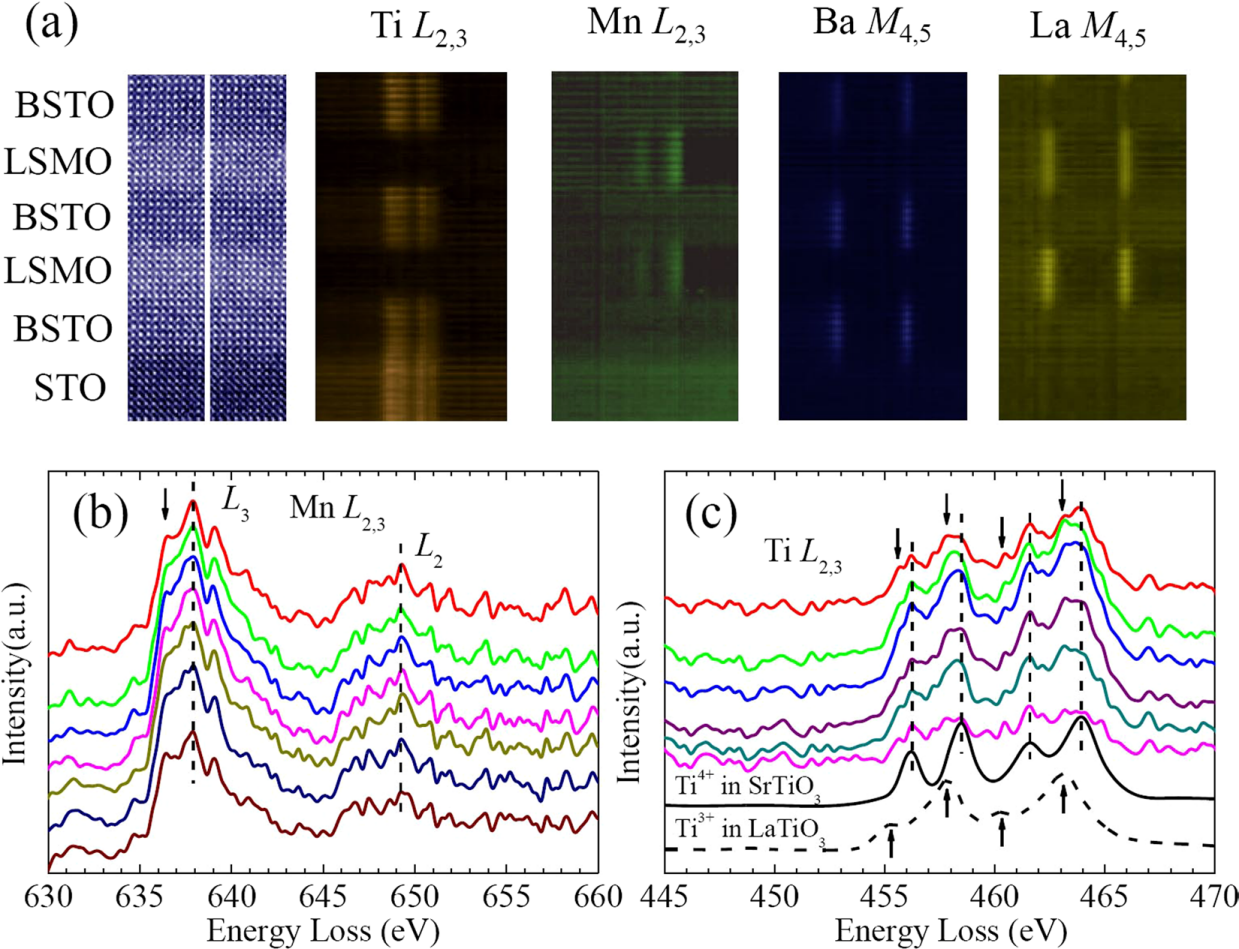
EELS spectra for the Ti-*L*_2,3_ and Mn-*L*_2,3_ edges of SL L7B6 grown on STO substrate. (**a**) The HAADF-STEM image marked with the position of the EELS line scan and EELS spectral maps for Ti-*L*_2,3_, Mn-*L*_2,3_, Ba-*M*_4,5_, and La- *M*_4,5_ edges. (**b**) Individual EELS spectra of Mn-*L*_2,3_ edges obtained from line scan across a layer of 7-u.c.-thick LSMO. Black arrow indicates an additional peak as a signatures of Mn^4+^ ions. (**c**) Individual EELS spectra of Ti-*L*_2,3_ edges obtained from line scan across a layer of 6-u.c.-thick BSTO. Reference spectra for Ti^4+^ and Ti^3+^ are also shown in (**c**), taken from the SrTiO_3_ and LaTiO_3_. Black arrows indicate the peak positions from the Reference Ti^3+^ EELS spectra, and black dashed lines indicate the peak positions from the Reference Ti^4+^ EELS spectra, respectively.

From the position of the fundamental diffraction peak of multilayers, the out-of-plane lattice constant *c* of these SLs, together with the bilayers and films, were calculated and shown in Fig. 1(b). The pseudo-cubic lattice constants of the bulk LSMO and BSTO are 3.876^28^ and 3.972 Å^29^, respectively, and that of the STO substrate is 3.905 Å. The lattice mismatch is −0.74% between LSMO and STO, 1.72% between BSTO and STO, and 2.48% between BSTO and LSMO, respectively. Therefore, the LSMO layers and film experience compressive stress along out-of-plane direction, and the BSTO layers and film experience the tensile stress along out-of-plane direction, indicating by red arrows in Fig. 2(a). Figure 3S exhibits an x-ray reciprocal space map of L14B12 around the STO (013) Bragg peak. Except the STO (013) peak (red arrow), the superlattice satellite peaks were readily apparent (white arrows). All the superlattice peaks are on the same *H* line with the substrate peak, indicating that the superlattices are under the fully coherent strain.

The out-of-plane lattice constant of the BSTO film is as large as 4.169 Å, a little larger than that (4.145 Å) of BSTO in LSMO/BSTO/STO bilayer. Both the out-of-plane lattice constants of the BSTO film and the BSTO layer in the LSMO/BSTO/STO bilayer are larger than that of the bulk BSTO due to the tensile stress along the out-of-plane direction. In contrast, the out-of-plane lattice constants of the LSMO film and the LSMO layer in LSMO/BSTO/STO bilayer are both smaller than that of the bulk LSMO due to the compressive stress in along the out-of-plane direction. The out-of-plane lattice constants of the LSMO/BSTO superlattice are determined by the out-of-plane cumulative stress (i.e. the substrate induced stress and interfacial stress). The out-of-plane lattice constants of the LSMO/BSTO superlattices are among 3.917 and 3.986 Å, tuned by both the thicknesses of the LSMO (t_LSMO_) and BSTO (t_BSTO_) layers, i.e., the out-of-plane lattice constants of the LSMO/BSTO superlattices slightly decrease with increasing the thickness ratio of t_LSMO_/t_BSTO_, or to say that an enhanced out-of-plane lattice constants for a superlattice are obtained with thicker BSTO layers or thinner LSMO layers.

### STEM and EELS

Atomic resolution characterization is crucial to investigate the interfacial structure and to demonstrate interfacial electronic states. This can be acquired by the state-of-the-art sophisticated aberration-corrected scanning transmission electron microscopy (STEM) using a high-angle annular dark-field (HAADF) detector in combination with electron energy loss spectroscopy (EELS). Figure 2(a) exhibits the schematic stacking sequence of LSMO/BSTO SLs grown on (001) STO substrates, with a total periodicity of 15. The stacking sequence of the LSMO/BSTO SL is shown in Fig. 2(b), showing a single supercell that is repeated the indicated number of times. The contrast of HAADF image exhibits ~*Z* ^1.7^ dependence, where *Z* is the atomic number^30^, therefore, the brighter bands corresponding to the nominal 7-u.c.-thick LSMO layers and the darker ones to the 6-u.c.-thick BSTO layers. A low-magnification cross-sectional HAADF-STEM image of L7B6 are shown in Fig. 2(c), clearly exhibiting well-defined superlattice structure consisting of 15 LSMO (bright) /BSTO (dark) bilayers, which is consistent with our design (as shown in Fig. 2(a)). The layers and interfaces are flat over long lateral distances. Higher magnification images allow the study of the interfacial structure in detail. High-resolution HAADF-STEM image is shown in Fig. 2(d). In Fig. 2(d), atomic columns of La(Sr), Mn, Ba(Sr), and Ti are resolved while O columns are not observed due to weak scattering of light atomic columns. The STEM image exhibits atomically sharp interfaces and coherent, which indicates the epitaxial growth of the LSMO/BSTO SLs. STEM imaging of SL L7B6 was also conducted under annular-bright-field (ABF) mode, as shown in Fig. 3S. The ABF-STEM images also exhibit atomically sharp interfaces and lateral coherency, indicating excellent epitaxial growth and high-quality of the LSMO/BSTO superlattices. Moreover, the epitaxial growth was also confirmed by the results of corresponding Fast Fourier transform (FFT) images (as shown in Fig. 5S).

The identification of the oxidation states of cations at atomic resolution combined with STEM has become a useful tool to identify the charge states in recent years^31–34^. EELS under STEM mode with high spatial resolution was used to examine the each element of individual atomic columns. EEL spectrum imaging was used to ascertain the interface structure and to probe any chemical disorder (e.g., interdiffusion). The Ti-*L*_2,3_, Mn-*L*_2,3_, Ba-*M*_4,5_, and La- *M*_4,5_ edges can be simultaneously recorded in a line scan across the interface of L7B6. Figure 3(a) exhibits the HAADF-STEM image marked with the position of the EELS line scan and EELS spectral maps for Ti-*L*_2,3_, Mn-*L*_2,3_, Ba-*M*_4,5_, and La- *M*_4,5_ edges across the STO substrate, three layers of 6-u.c.-thick BSTO, and two layers of 7-u.c.-thick LSMO. From the EELS spectral maps it can be concluded that the interfaces are very sharp and little chemical interdiffusion is present. EELS line scans were used to characterize not only the cations content profiles but also the local Mn and Ti valence along the thickness of LSMO/BSTO superlattices. Figure 3(b) shows the Mn *L*_2,3_ edges from the layer of 7-u.c.-thick LSMO. The spectra of the Mn *L* edges do not exhibit strong outstanding features as one might expect for these spectroscopic features dominated by multiplet effects^31^. The line shape of the spectrum depends strongly on the multiplet structure given by the Mn 3*d*-3*d* and 2*p*-3*d* Coulomb and exchange interactions, as well as by the local crystal fields and the hybridization with the O 2*p* ligands. It can be seen from Fig. 3(b) that no resolvable shifts on the Mn-*L-*edge fine structures among the Mn-O planes of the LSMO layer can be resolved, which is generally expected when the local Mn valence changes^9, 35^. No obvious chemical shift of the Mn *L*_3_ peak was also observed in previous work on LaMnO_3_-SrMnO_3_ superlattice samples^36, 37^. It was proposed if the Mn concentration was constant in their LMO-SMO superlattice, the lack of a chemical shift has been suggested to result from either oxygen vacancies in the LSMO film or the covalent Mn-O bond rather than a purely ionic bond^37, 38^. An additional peak about 1.5 eV below the main *L*_3_ peak can be seen, which is as a signature of Mn^4+^ ion^39^. It can be noted that the additional peaks (indicated by the black arrow) of the end interfacial MnO_2_ planes show a slight enhancement, which can be ascribed to a slightly increased concentration of Mn^4+^ ion in the two end interfacial MnO_2_ planes.

For investigating the variation of the valence state of Mn, except for the change of the intensity of the shoulder at higher energy corresponding to Mn^4+^, the ratio of the integrated intensity of the Mn *L*_3_ and *L*_2_ lines (*L*_23_ ratio) has been used to quantify the Mn valence state^39^. The Mn EELS data close to the LSMO/BSTO interface is shown in Fig. 6S(a). After scaling and subtraction of a Shirley function (black dotted line), the remaining signals under the *L*_3_ and *L*_2_ lines are integrated, and their ratio (*L*_23_ ratio) is calculated. The calculated *L*_23_ ratio is about 2.64. The nominal Mn oxidation state for our La_0.7_Sr_0.3_MnO_3_ is +3.3. According the Varela’s results, the *L*_23_ ratio of the nominal Mn oxidation state for +3.3 is about 2.5, and the larger the Mn *L*_23_ ratio, the smaller the Mn oxidation state is. The smaller Mn oxidation state should be attributed to the oxygen vacancies present in the LSMO layers. Figure 6S(b) shows the dependence of the calculated *L*_23_ ratio with the distance of LSMO layer from the interface. It can be seen from Fig. 5S(b) that the *L*_23_ ratio close to the interfaces are a little smaller than that inside MnO_2_ layers, indicating the Mn valence state close to the interfaces is a little higher than that inside MnO_2_ layers.

**Figure 4:**
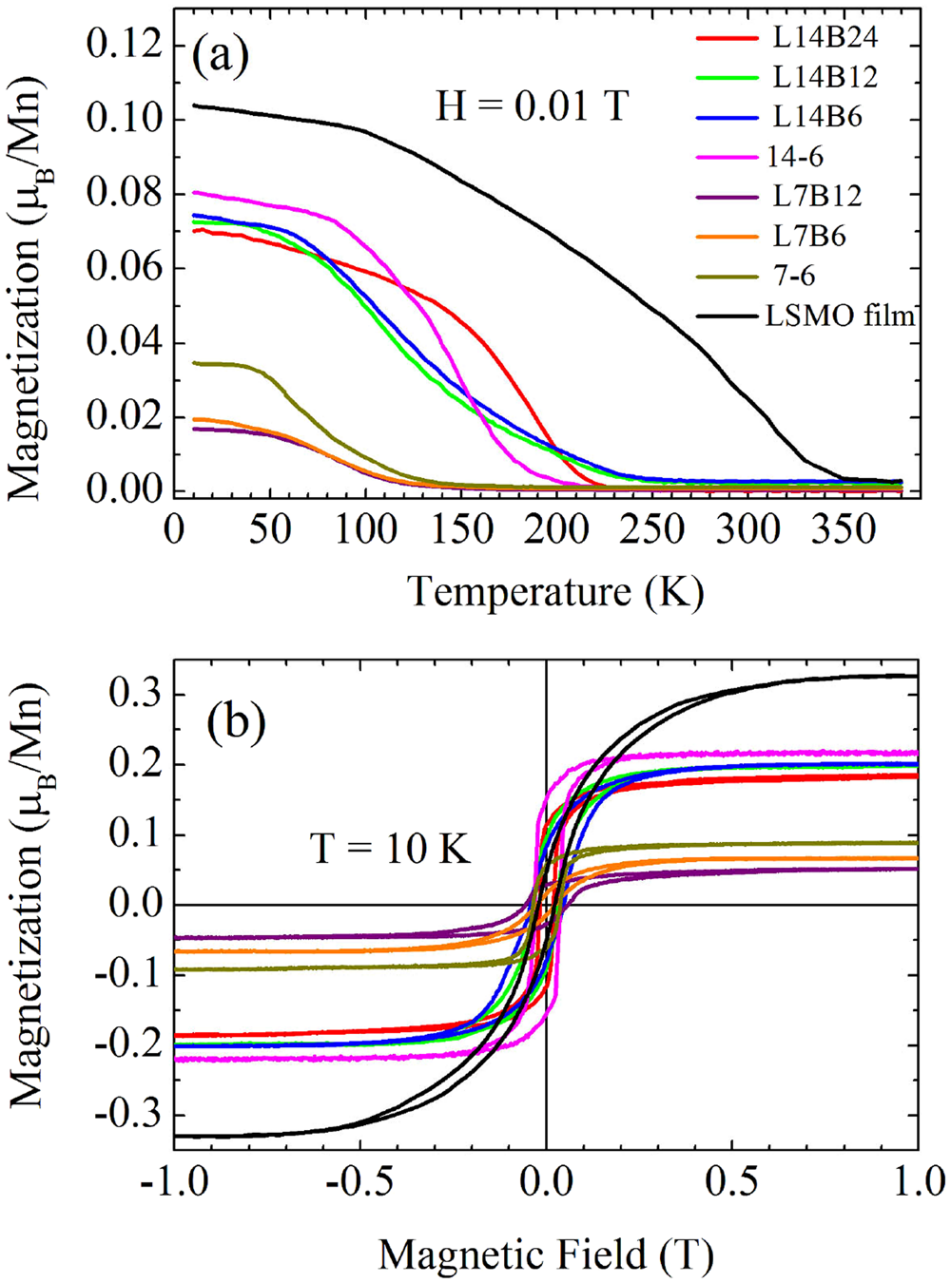
(**a**) Magnetization as a function of temperature for the LSMO/BSTO SL series and LSMO film. Magnetization was measured at field-cooled mode in a magnetic field of 0.01 T. (**b**) Magnetization as a function of field for the LSMO/BSTO SL series and LSMO film at 10 K. Magnetization is normalized by the volume of the LSMO layer.

Individual EELS spectra of Ti-*L*_2,3_ edge obtained from line scan cross the 6-u.c.-thick BSTO layer of are shown in Fig. 3(c). Unlike Mn, there is an empty *d* orbital for Ti^4+^ while there is one *d* electron in *d* orbital for Ti^3+^, and the EELS spectra are quite different for Ti^4+^ and Ti^3+^ with large shift and different line shapes, The reference EELS spectra for Ti^4+^ and Ti^3+^ are taken from the SrTiO_3_ and LaTiO_3_. It can be seen from Fig. 3(c) that every Ti-*L*_2,3_ EELS spectroscopic features are the mixture of those from Ti^4+^ (black dashed lines) and from Ti^3+^ (black arrows), indicating that the Ti sites exhibit mixed valence between 3+ and 4+. The spectral shape of the Ti EELS changes very tiny across the entire layers, unlike the case of the Mn EELS. This difference maybe come from the different mechanism of the electrons transferring between the *t*_2*g*_^0^/*t*_*2g*_^1^ orbitals of the Ti^4+^/Ti^3+^ ions and the *t*_2g_^3^ *e*_g_^0^/*t*_2g_^3^ *e*_g_^1^ orbitals of the Mn^4+^/Mn^3+^ ions, and the *t*_*2g*_^0^/*t*_*2g*_^1^ orbital configuration is more efficient electron transfer. The presence of Ti^3+^ features at interface should be caused by an electron transfer to Ti^4+^ ion across the interfaces, since the valence of Ti in the stoichiometric BSTO is +4. Such charge-transfer mechanisms across the oxide interface were also occurred at the other oxide interfaces of the heterostructures and superlattices evidenced by the STEM-EELS spectra, and the typically range is within ~1 nm of the interface^2, 33, 34, 36^. Combination of the EELS analysis of Mn and Ti, an unambiguous charge transfer involving an electron transferring from the LSMO layers to BSTO layers (Mn^3+^→Mn^4+^; Ti^4+^→Ti^3+^) across the interface are confirmed. Finally, both the oxidization states of the Mn and Ti ions should be changed due to the existence of the oxygen vacancies. The existence of the oxygen vacancies in the LSMO layers were confirmed by the analysis of the EELS data, as shown in Fig. 4S. Both the oxidization states of the Mn and Ti ions should be changed due to the existence of the oxygen vacancies.

Moreover, from the results of HR-STEM and EELS (Figs 2(d) and 3(a)) it can be confirmed that the atomic stacking sequences of the interfaces of LSMO/BSTO and BSTO/LSMO are MnO_2_/La(Sr)O/TiO_2_/Ba(Sr)O and TiO_2_/Ba(Sr)O/MnO_2_/La(Sr)O, respectively.

### Magnetic Properties

Charge distribution at the interfaces of the LSMO/BSTO SLs could be expected to have a large impact on the transport and magnetic properties of the LSMO/BSTO SLs. In order to understand these, transport and magnetic measurements were performed using PPMS. Figure 4 exhibits the magnetization of the LSMO/BSTO SLs along with comparison with the LSMO film as a function of temperature and magnetic field (*M*(*T*)). The magnetization is normalized by the volume of the LSMO layer. Magnetization was measured at field-cooled mode in a magnetic field of 0.01 T. It can be seen from Fig. 4(a) that the magnetizations of all LSMO/BSTO SLs exhibit paramagnetic-to-ferromagnetic transition with decreasing temperature, in accordance with the behavior of the temperature-dependent magnetization of the single-layer LSMO film. The ferromagnetic (FM) properties of all LSMO/BSTO SLs at low temperature were confirmed by exhibiting ferromagnetic hysteresis loops at low temperature, as shown in Fig. 4(b). Table 1 shows the dependences of paramagnetic-to-ferromagnetic transition temperature (*T*_*C*_), the saturated magnetization moment (*M*_*S*_) under 1 T at 10 K, and corrective field (*H*_*C*_) with the thicknesses of the LSMO layer in the LSMO/BSTO SLs. It can be seen from Fig. 4 and Table 1 that the *M*(*T*) curves of LSMO/BSTO SLs can be divided into two groups according to the magnitude of magnetization and *T*_*C*_. One group includes the LSMO/BSTO SLs with the LSMO layer thickness of 7 monolayers (MLs), of which the saturated magnetization moment (*M*_*S*_) under 1 T at 10 K is about 0.05–0.09 μ_B_/Mn and *T*_*C*_ is about 60–70 K. The other are the LSMO/BSTO SLs with the LSMO layer thickness of 14 MLs, of which *M*_*S*_ is about twice of the group one (at under 1 T at 10 K about 0.18–0.22 μ_B_/Mn) and *T*_*C*_ among 110–180 K.

**Table 1:** The dependences of *T*_*C*_, *M*_*S*_, and *H*_*C*_ with the thicknesses of the LSMO layer in the LSMO/BSTO SLs.

Thickness of the LSMO layer (u.c.)	SL No.	*T*_*C*_	*M*_*S*_	*H*_*C*_
		(K)	(μ_B_/u.c.)	(Oe)
7	L7B6 layer	58	0.089	361
7	L7B6	83	0.067	243
7	L7B12	89	0.049	565
14	L14B6 layer	145	0.217	341
14	L14B6	112	0.201	443
14	L14B12	104	0.198	325
14	L14B24	119	0.189	185
LSMO thin film	LSMO	285	0.326	219

### Resistivity and MR

**Figure 5:**
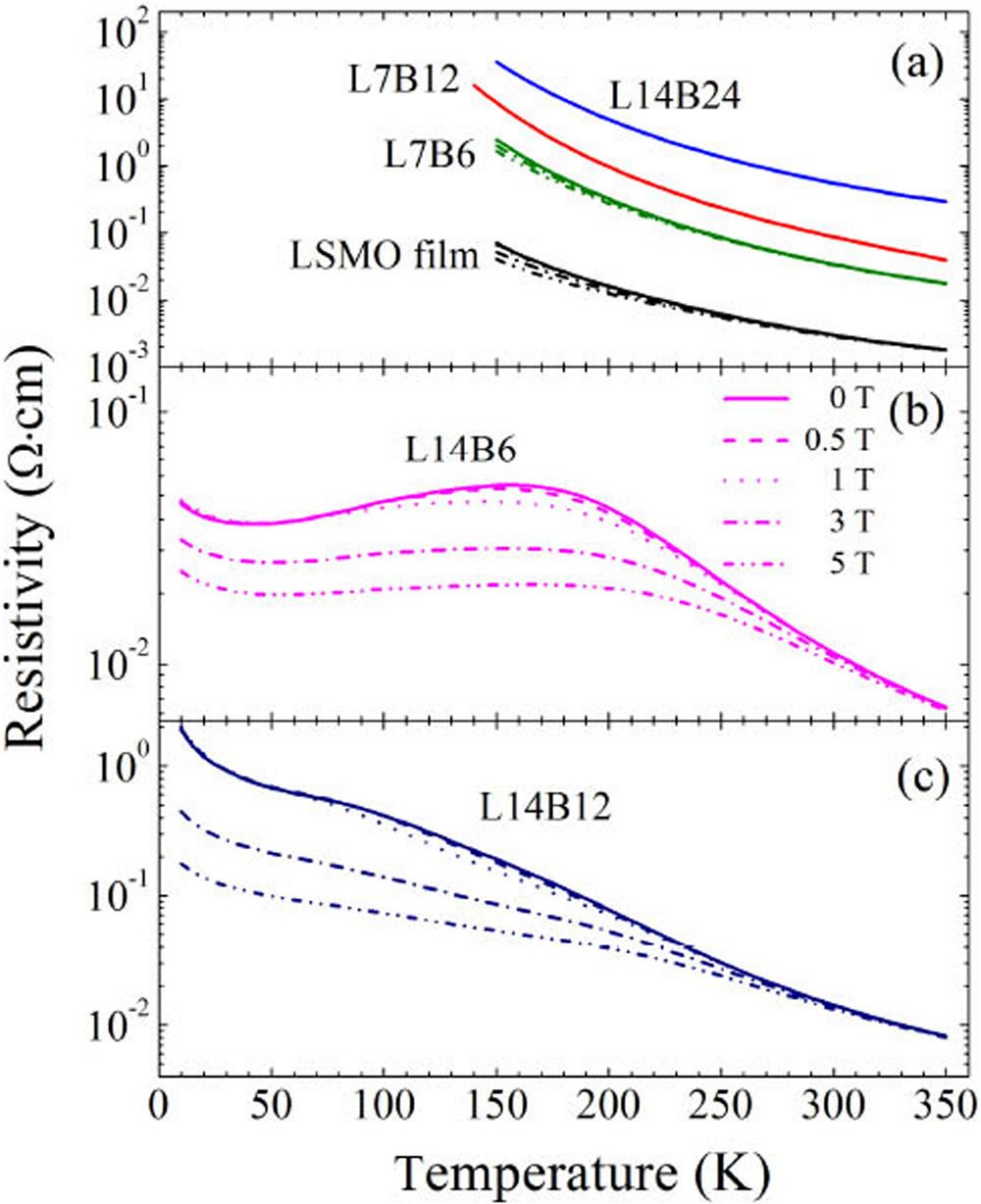
(**a–c**) Resistivity as a function of temperature for the superlattices and LSMO film in applied fields of 0, 0.5, 1, 3, 5 T.

The resistivity, ρ, of each superlattice is calculated from its resistance using the simplified assumption that the current flows only in the LSMO layers since the resistivity of BSTO is orders of magnitude higher than that of LSMO and the entire stack rather than only the LSMO layers contribute to the conduction. The temperature dependence of the resistivity of the superlattices and the LSMO film under the magnetic fields of 0, 0.5, 1, 3, 5 T are presented in Fig. 5(a–c). The LSMO film exhibits insulating characteristics with a large negative MR which monotonically increases in magnitude with decreasing temperature, and the resistivity at lower temperature is above the instrumental limit, as shown in Fig. 5(a). The similar transport and MR behaviors have been observed in ultrathin LSMO films^40^ as well as LSMO films with oxygen vacancies grown under low oxygen pressures^41^. Since the thickness of the LSMO film is 130 nm, the oxygen vacancies should play the key role in the strong depression of the double-exchange magnetotransport properties in the LSMO film. The SLs of L14B24, L7B12, and L7B6 exhibit similar insulating behaviors as the LSMO film across the entire measurable temperature range, as shown in Fig. 5(a). The resistivity ρ of the SLs increases with increasing the thickness of the BSTO layers, and larger than that of the LSMO film. This indicates that the thickness of the BSTO layer plays a determining role in the transport properties of the system, since the BSTO layers act as barrier layers to prevent electron hopping through BSTO layers.

Quite different from other LSMO/BSTO SLs, SL L14B6 displays a transition from semiconducting to metallic behavior at 160 K, followed by an increase for temperatures below 40 K, as shown in Fig. 5(b). This qualitative behavior is also observed in low Sr-doped La_1−x_Sr_x_MnO_3_ which is attributed to the weak localization of carriers due to disorder^42^, and in ultrathin LSMO films which is attributed to the presence of quantum interference effects due to weak localization^43^. SL L14B12 also displays similar features, as shown in Fig. 5(c), although with much larger resistivity and lower transition temperature.

**Figure 6:**
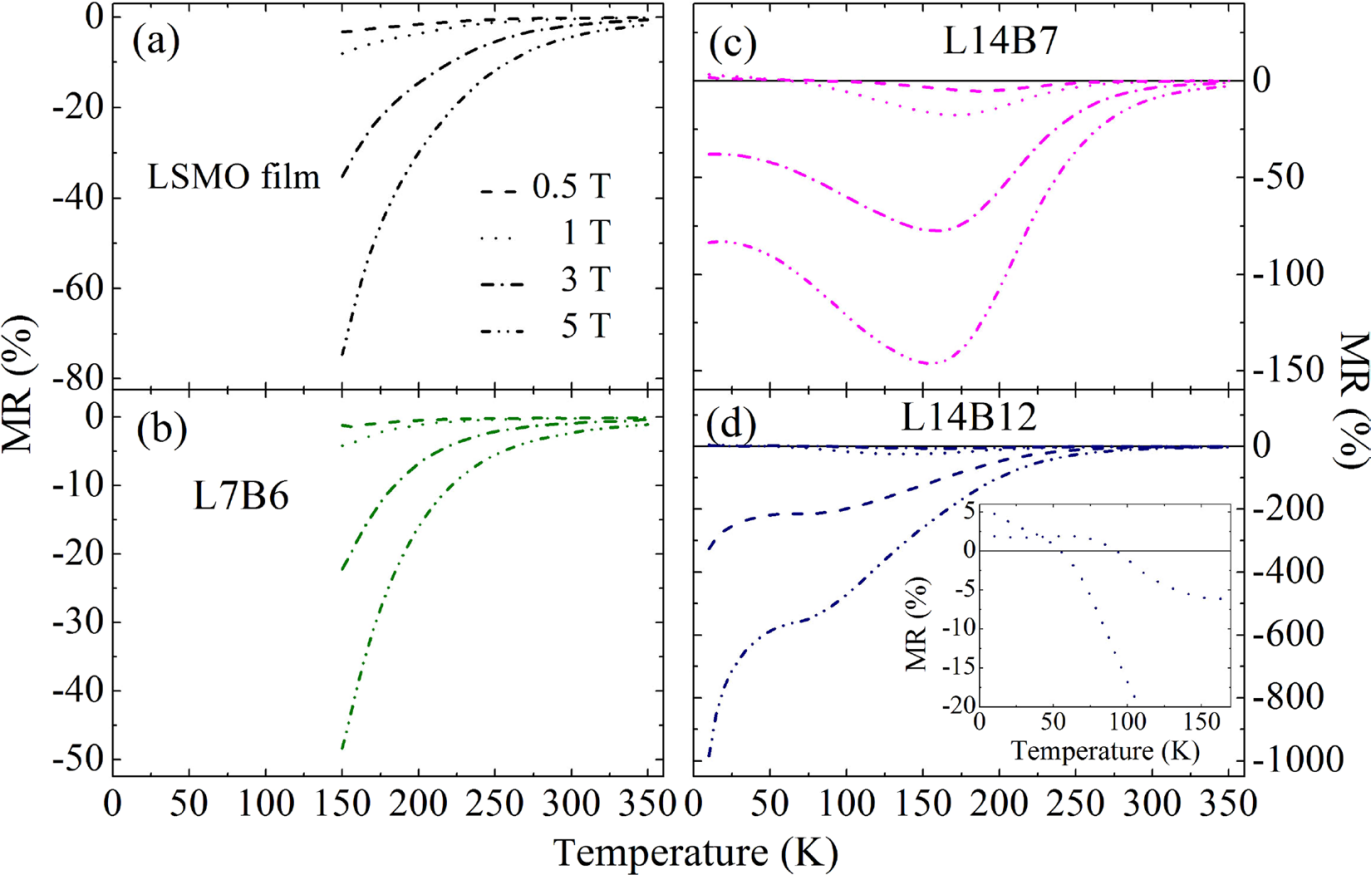
Magnetoresistance, MR = [ρ(H)−ρ(0)]/ρ(H) × 100 as a function of temperature for (**a**) the LSMO film, (**b**) L7B6, (**c**) L14B6, and (**d**) L14B12.

Magnetoresistance, MR = [ρ(H)−ρ(0)]/ρ(H) × 100, as a function of temperature for the LSMO/BSTO SLs and the LSMO film are shown in Fig. 6. MR of SL L7B6 shows similar features as that of the LSMO film, exhibiting negative MR at the measureable temperature range. However, SL L14B6 shows a negative peak in the magnetoresistance at the temperature close to a transition from semiconducting to metallic behavior, and large MR extends over low temperatures. Especially in SL L14B12, MR increases with temperature and huge MR subsist to low temperature, as −1000% under 5 T at 10 K. These results are obviously different from those of LSMO bulk, which exhibit negligibly small MR at low temperature far below *T*_*C*_, because the local moments within each magnetic domain orientate rather unanimously and the suppression of the local moment fluctuations by a magnetic field can be neglected^21–23, 42^. In additional, an anomalous positive MR is observed both in SLs L14B6 and L14B12 at low temperature and under low magnetic fields, seen in the inset of Fig. 6(d).

## Discussion

The lattice parameters of the LSMO/BSTO SLs are determined by the interfacial strain, tuned by both the thicknesses of the LSMO (t_LSMO_) and BSTO (t_BSTO_) layers, i.e., the out-of-plane lattice constants of the LSMO/BSTO superlattices slightly decrease with increasing the thickness ratio of t_LSMO_/t_BSTO_, or to say that an enhanced out-of-plane lattice constants for a superlattice are obtained with thicker BSTO layers or thinner LSMO layers. On the other hand, an unambiguous charge transfer involving an electron transferring from the LSMO layers to BSTO layers (Mn^3+^→Mn^4+^; Ti^4+^→Ti^3+^) across the interface are confirmed by EELS. All above observed phenomena indicate that the interfacial effects play a crucial role on the structural and electronic properties of the LSMO/BSTO SLs.

Moreover, the interfacial effects also affect the transport and magnetic properties of the LSMO/BSTO SLs. The interfacial effects cause suppression of the FM state of the LSMO/BSTO SLs. The results of the magnetic property measurements indicate that the overall magnetization is dominated by the ferromagnetic LSMO layers. It is also worth noting that the magnetization of the LSMO/BSTO bilayer is larger than that of LSMO/BSTO SLs with the same thickness of the LSMO monolayer. Since the magnetizations of LSMO/BSTO SLs are normalized by the volume of the LSMO layer, the normalized magnetizations of LSMO/BSTO SLs should be same if there are no any interfacial effects. The magnetic profiles would be modulated by the interfaces, i.e., the presence of spin frustration at the interfaces or phase separation^44, 45^. Finite-size effects in the superlattices will decrease the long-range interactions, and cause the rapid decrease of *T*_*C*_ and magnetization with decreasing the LSMO layer thickness. Another reason may come from the interfacial orbital-lattice coupling induced by the biaxial lattice strain, which tends to increase the in-plane Mn-O bond length, reducing the effective electron hopping and reducing the effect of the double exchange mechanism^46, 47^. It should be noted that a significant reduction can be seen both in the magnitude of magnetization and *T*_*C*_ of all of the LSMO/BSTO SLs compared with the single-layer LSMO film, indicating that all of the LSMO/BSTO SLs present signatures of the important interfacial effects.

The oxygen vacancies always play a key role in the strong depression of the double-exchange magnetotransport properties in the manganite oxides^40, 41^, therefore the oxygen vacancies should also affect the magnetotransport properties of the LSMO/BSTO SLs. The effects of the oxygen vacancy on the magnetotransport properties are as following. Firstly, the local Jahn-Teller effect is enhanced by oxygen vacancies, and the strong interaction between the electron and the Jahn-Teller lattice distortion is generally regarded as one main reason for the insulating state of LSMO film. Secondly, the oxygen vacancies break the octahedron symmetry, therefore the energy of the *e*_*g*_ orbital pointing at the oxygen vacancy is reduced, which introduces a strong electron-lattice interaction and tends to localize the electrons so that the superexchange tends to dominate the magnetic interaction. Thirdly, the oxygen vacancies lead to the local distortion and rotation of the oxygen octahedrons, which leading to decrease the Mn-O-Mn bond angle and consequently the probability of hopping between Mn ions. Finally, the oxygen vacancies also introduce disorder. Except for the oxygen vacancies, the interface effects are believed to be response to the strong depression of the magnetotransport properties of these SLs. These interface effects include charge redistribution at the interface triggered by a polarity discontinuity^1, 4^, and the STEM-EELS results confirmed an unambiguous charge transfer involving an electron transferring from the LSMO layers to BSTO layers (Mn^3+^→Mn^4+^; Ti^4+^→Ti^3+^) across the interface. Another is a preferential out-of-plane 3*d e*_*g*_ (3*z* ^2^−*r* ^*2*^) Mn orbital occupation, attributed to symmetry breaking at the interfaces^48^.

The magnetoelectric coupling associated with the multilayers of magnetostrictive and piezoelectric perovskite oxides could attribute to these effects^14–19, 49^. The electric field can be generated by ferroelectric BSTO layers in the superlattice structure by producing the charges at the interface, which in turn changes the resistance of LSMO layer. Usually negative colossal MR is observed in manganite bulks and thin films arising from the magnetic field suppressing the scattering of carriers by localized magnetic moments^21–23, 42^. Similar phenomenon was also been observed in La_0.7_Pb_0.3_MnO_3_ bulk^48^ and in the La_0.9_Sr_0.1_MnO_3_/SrNb_0.01_Ti_0.09_O_3_ heterostructure^50–52^, which could be attributed to quantum interference effects due to the Coulomb interactions between carriers enhanced by disorder and the interface effect. The charge and spin states are reconstructed at the interfaces in our system and hence affect the electronic and magnetic properties of the entire system, which should be affected the observed positive MR.

In summary, an epitaxial growth with sharp interfaces between LSMO and BSTO layers was confirmed by STEM and SXRD. An unambiguous charge transfer involving an electron transferring from the LSMO layers to BSTO layers (Mn^3+^→Mn^4+^; Ti^4+^→Ti^3+^) across the interface were resolved by EELS. These observations are attributed to the possible modification in the stereochemistry of the Ti and Mn ions in the interfacial region. The out-of-plane lattice parameter, Curie temperature, and magnetoresistance are strongly affected by sublayer thickness. Huge magnetoresistance subsisting to low temperature is also observed in the LSMO/BSTO superlattices. All spectral changes identified at a nanometer scale and their potential effect on the degradation of magnetic and transport properties at a macroscopic level. These findings highlight the importance of dependence on sublayer thickness and temperature, illustrating the high degree of tenability in these artificially layered materials.

## Methods

### Fabrication

LSMO/BSTO superlattices were grown on STO single crystals by a laser molecular-beam epitaxy system (Laser-MBE) combined with a reflected high energy electron diffraction (RHEED). The LSMO/BSTO superlattices were deposited at 590 °C using a XeCl excimer laser (308 nm, 1.5 J/cm^2^, 2 Hz) at oxygen partial pressure of 5 Pa, which represented the best compromise for both obtaining the smooth interfaces and surfaces and high-quality of the LSMO and BSTO layers. The LSMO/BSTO superlattices composed of individual LSMO layers with 7 and 14 u.c. and BSTO layer thickness of 6, 12, and 24 u.c. with a total periodicity of 15 were realized. For comparison, the LSMO and BSTO thin films and LSMO/BSTO bilayer were also grown at the same conditions. RHEED oscillations were observed and were used to calibrate the number of layers grown. After growth, all samples were *in-situ* annealed for 30 minutes.

### SXRD and RSM

The epitaxial nature and structural characterization of the LSMO/BSTO superlattices were identified by high-resolution SXRD and x-ray reciprocal space map (RSM) at the BL14B1 beam line of Shanghai Synchrotron Radiation Facility (SSRF), using a 1.24 Å X-rays with a Huber 5021 six-axis diffractometry.

### STEM and EELS

To characterize the atomic resolution characterization of the LSMO/BSTO superlattices, state-of-the-art sophisticated aberration-corrected STEM using a HAADF detector were utilized. The HAADF imaging was executed using an ARM-200F (JEOL, Tokyo, Japan) STEM operated at 200 kV with CEOS Cs corrector (CEOS GmbH, Heidelberg, Germany) to cope with the spherical aberration of the probe-forming condenser lens. More detailed chemical composition and bonding was probed on an atomic scale using spatially resolved EELS.

### Magnetization and resistivity measurements

Temperature dependences of magnetization, resistivity, and MR of the films and LSMO/BSTO superlattice were performed using a Physical Properties Measurement System (PPMS, Quantum Design Inc.). The standard four-point probe method was used to measure the film resistivity versus temperature (*ρ(T)*). The magnetotransport properties as a function of temperature and magnetic field were measured by a standard four-point probe method using PPMS.

## Electronic supplementary material

Supporting information
