# Supplementary material for: Manipulating magnetoelectric properties by interfacial coupling in La0.3Sr0.7MnO3/Ba0.7Sr0.3TiO3 superlattices
Source: Sci Rep. 2017 Aug 9;7:7693. doi: 10.1038/s41598-017-08260-y (PMC5550473; doi:10.1038/s41598-017-08260-y)
Supplement: Supplementary file 1 — Supporting information [file 41598_2017_8260_MOESM1_ESM.doc]

**Manipulating magnetoelectric properties by interfacial coupling in La_0.3_Sr_0.7_MnO_3_/Ba_0.7_Sr_0.3_TiO_3_ superlattices**

Haizhong Guo^1,6*^, Qingqing Li^1,2^, Zhengzhong Yang^1^, Kui-juan Jin^1,3,4,*^, Chen Ge^1^, Lin Gu^1^, Xu He^1^, Xiaolong Li^5^, Ruiqiang Zhao^1^, Qian Wan^1^, Jiesu Wang^1^, Meng He^1^, Can Wang^1^, Huibin Lu^1^, Yuping Yang^2^ & Guozhen Yang^1, 2^

*^1^Institute of Physics, Chinese Academy of Sciences, Beijing 100190, China*

*^2^School of Science, Minzu University of China, Beijing 100081, China*

*^3^Collaborative Innovation Center of Quantum Matter, Beijing, China*

*^4^University of Chinese Academy of Sciences, Beijing 100049, China*

*^5^Shanghai Synchrotron Radiation Facility (SSRF), Shanghai Institute of Applied Physics, Chinese Academy of Sciences, Shanghai 201204, China*

| *^6^School of Physical Engineering, Zhengzhou University, Zhengzhou, Henan 450001, China*  To investigate the epitaxial nature and crystallinity, SXRD were performed on various samples, including the LSMO/BSTO superlattices, the LSMO and BSTO films, and the LSMO/BSTO/STO bilayers. Figure 1S shows SXRD patterns of the LSMO/BSTO superlattices, the LSMO and BSTO films, and the LSMO/BSTO/STO bilayer. It can be seen from Figure 1S that all samples show only the (00l) Bragg reflections, and no diffraction peaks from secondary phase or randomly oriented grains are observed, indicating that all the films, bilayers, and superlattice were epitaxially grown along the *c*-axis orientation with a good single phase.  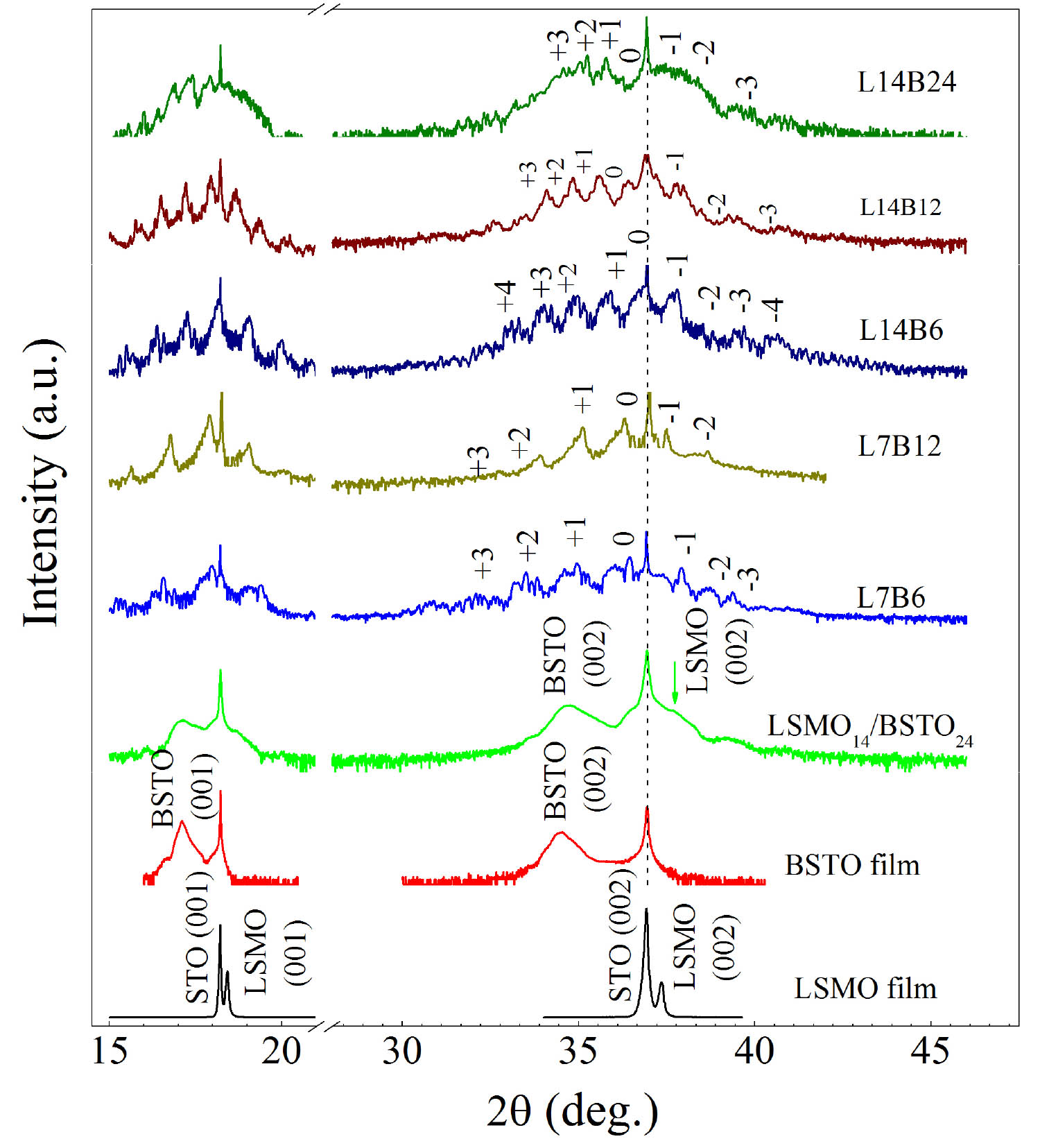  *Figure 1S (Color online) SXRD θ-2θ scan curves of the LSMO/BSTO SL series, BSTO/LSMO bilayer, BSTO film, and LSMO films grown on STO substrates, respectively. The numbers 1, 2, 3,…indicate the order of the satellite peaks.*  X-ray φ scan was carried out to confirm the epitaxial growth of SLs. In x-ray φ scan measurements, (202) plane of L14B12 was selected. The (202) φ scan result was obtained by rotating the sample 0-360° around the surface normal. This result is plotted in Fig. 2S. In Fig. 2S, four equally spaced peaks separated by 90° were observed. Therefore, Fig. 2S clearly indicates that L14B12 was epitaxially grown on the ST0 substrate. Moreover, the SL peaks are the same with respect to the substrate peaks, suggesting the epitaxial relationships: [001]SL//[001]STO and [100]SL//[100]STO.  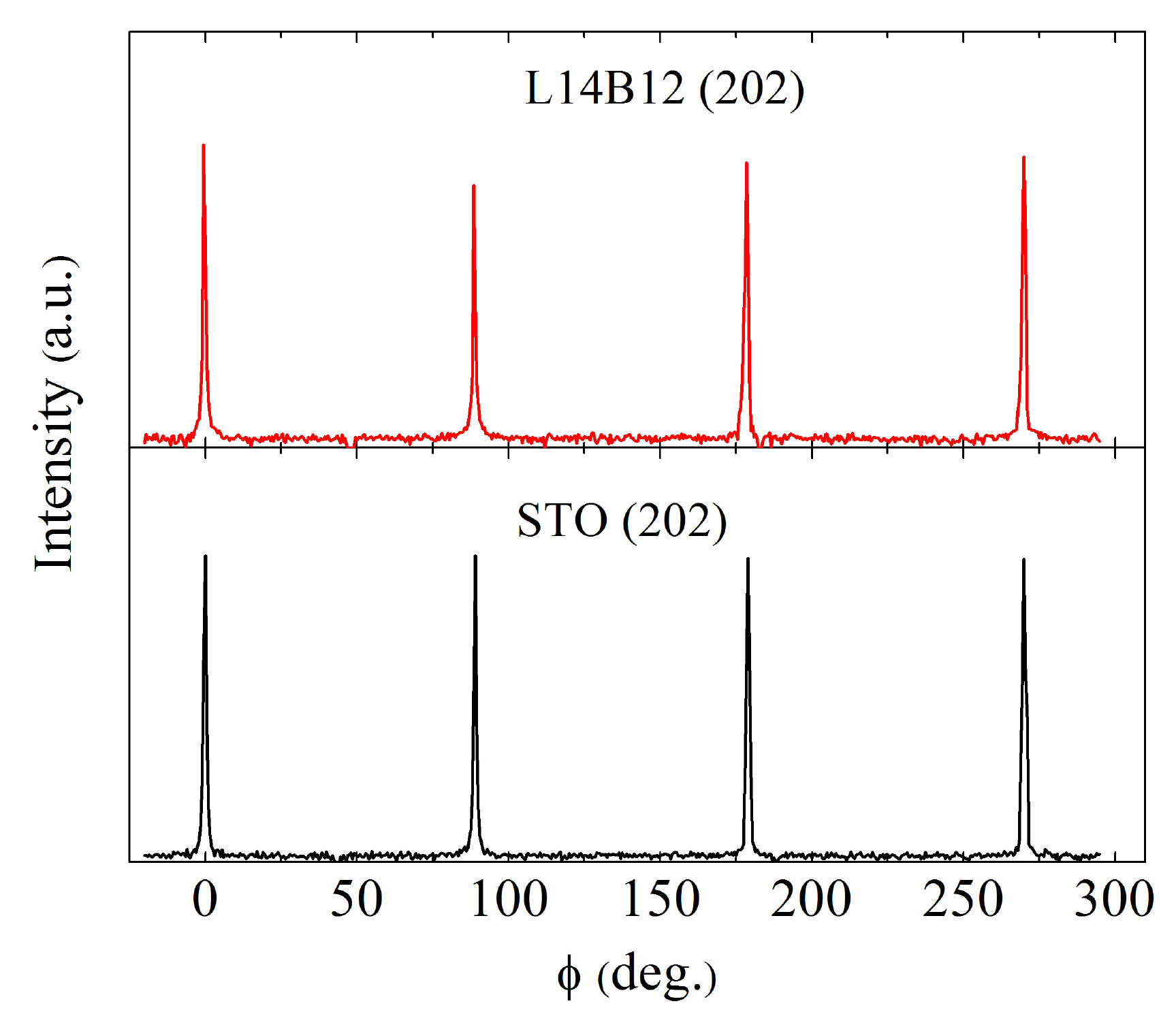  *Figure 2S φ scans of the (202) reflections of (a) SL L14B12 and (b) the STO substrate, respectively.*  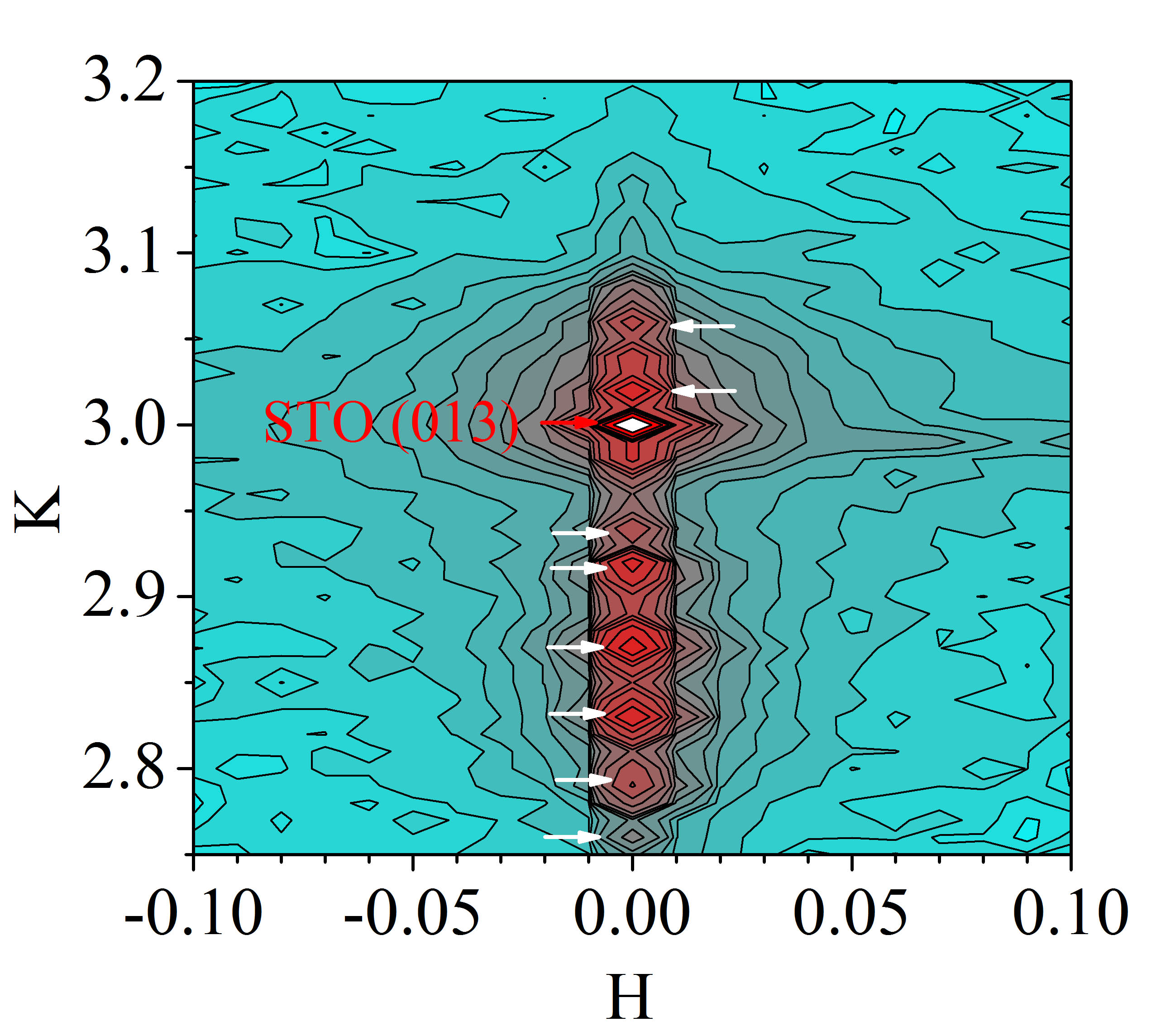  *Figure 3S X-ray reciprocal space map around the substrate STO (013) bragg peak recorded from SL L14B12. Well-defined superlattice satellite peaks are clearly observed, confirming the high-quality of the superlattices.*  Figure 3S exhibits an x-ray reciprocal space map of L14B12 around the STO (013) Bragg peak. Except the STO (013) peak (red arrow), the superlattice satellite peaks were readily apparent (white arrows). All the superlattice peaks are on the same *H* line with the substrate peak, indicating that the superlattices are under the fully coherent strain.  STEM imaging of SL L7B6 was also conducted under annular-bright-field (ABF) mode, as shown in Figure 4S. The ABF-STEM images also exhibit atomically sharp interfaces and lateral coherency, indicating excellent epitaxial growth and high-quality of the LSMO/BSTO superlattices. |
| --- |


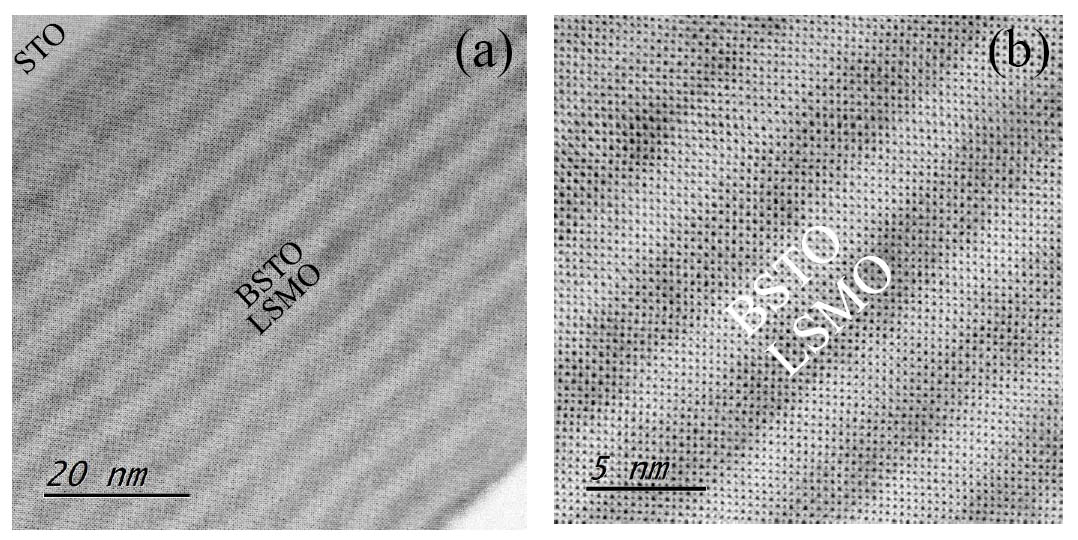


*Figure 4S (Color online) (a) A low-magnification cross-sectional ABF-STEM image and (b) High-resolution ABF-STEM image of the SL L7B6.*

The fast Fourier transform (FFT) was also used to confirm the epitaxial growth of the LSMO/BSTO SL. The FFT patterns taken from the different areas of SL L7B6 marked by the corresponding color boxes of the HAADF-STEM image in Figure 5S (a) were shown in Figure 5S (b)-(d), respectively. FFT images of the STO substrate and the LSMO/BSTO are almost the same, indicating the epitaxial relationship of the STO substrate and the LSMO/BSTO SLs.


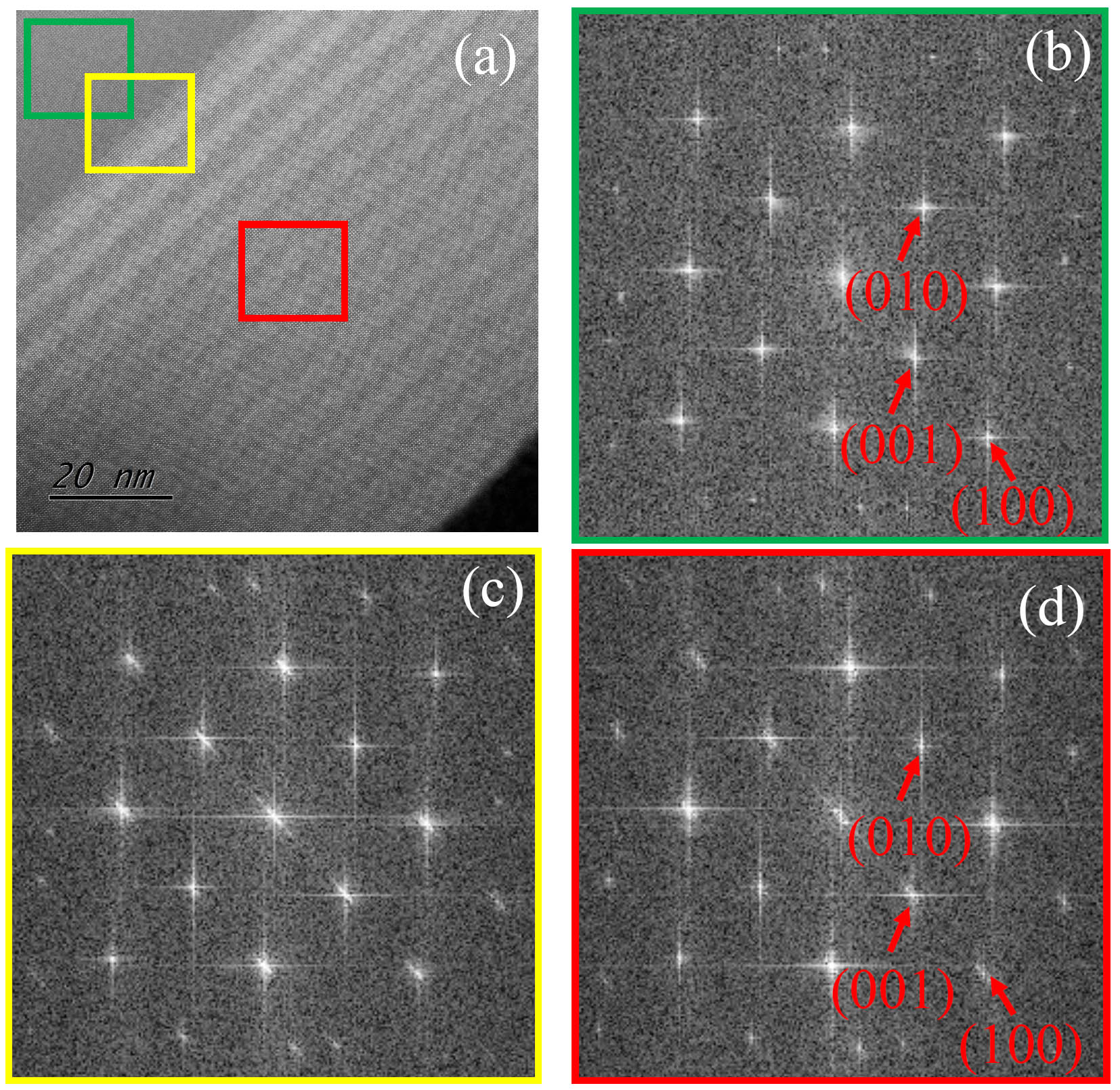


*Figure 5S (Color online) (a) Low-magnification cross-sectional HAADF-STEM image of SL L7B6 along the [100] axis of STO. (b) The FFT pattern taken from the area in the STO substrate marked by the blue box in (a). (c) The FFT pattern taken from the area across the interface marked by the yellow box in (a). (d) The FFT pattern taken from the area in the LSMO/BSTO SL marked by the red box in (a).*

We tried to quantify the valence state of Mn using EELS data. Figure 6S(a) shows the Mn EELS data close to the LSMO/BSTO interface. After scaling and subtraction of a Shirley function (black dotted line), the remaining signals under the *L*_3_ and *L*_2_ lines are integrated, and their ratio (*L*_23_ ratio) is calculated. The calculated *L*_23_ ratio is about 2.64. The nominal Mn oxidation state for our La_0.7_Sr_0.3_MnO_3_ is +3.3. According the Varela’s results (Ref. 1), the *L*_23_ ratio of the nominal Mn oxidation state for +3.3 is about 2.5, and the larger the Mn *L*_23_ ratio, the smaller the Mn oxidation state is. The smaller Mn oxidation state should be attributed to the oxygen vacancies present in the LSMO layers. Figure 6S(b) shows the dependence of the calculated *L*_23_ ratio with the distance of LSMO layer from the interface. It can be seen from Figure 6S(b) that the *L*_23_ ratio close to the interfaces are a little smaller than that inside MnO_2_ layers, indicating the Mn valence state close to the interfaces is a little higher than that inside MnO_2_ layers.


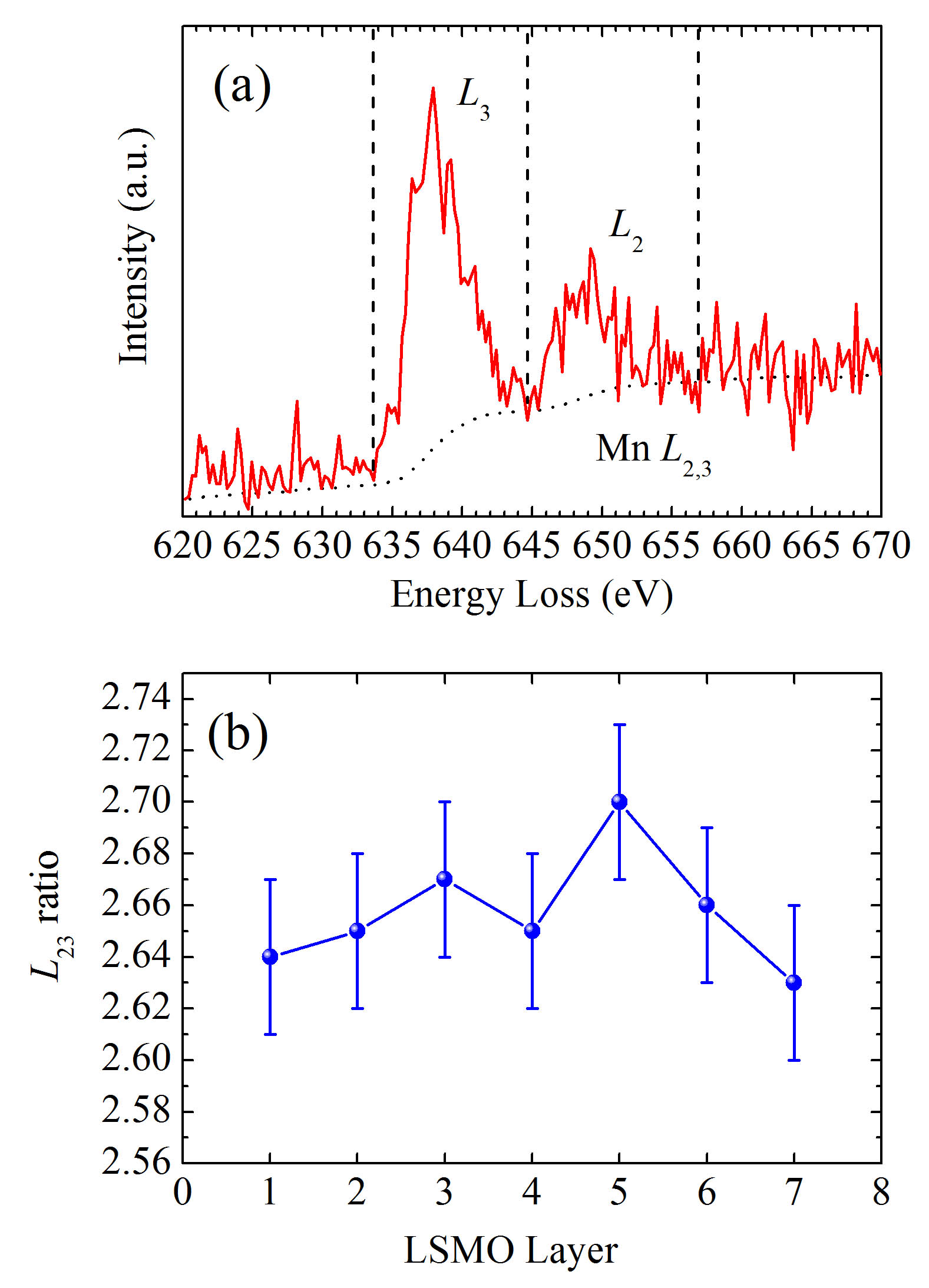


*Figure 6S. (Color online) (a) Sketch showing a generic Mn L_2,3_ edge of the LSMO layer close to the LSMO/BSTO interface and the approximate position of the windows used for integration of the L_3_ and L_2_ line intensities. After scaling and subtraction of a Shirley function (black dotted line), the remaining signals under the L_3_ and L_2_ lines are integrated, and their ratio (L_23_ ratio) is calculated. (b) Dependence of the L_23_ ratio with the distance of LSMO layer from the interface.*

Reference:

1. Varela, V. *et al.* Atomic-resolution imaging of oxidation states in manganites, *Phys. Rev. B* **79**, 085117 (2009).
